# Supplementary material for: Dys-R Questionnaire: A Novel Screening Tool for Dysbiosis Linked to Impaired Gut Microbiota Richness
Source: Nutrients. 2023 Oct 5;15(19):4261. doi: 10.3390/nu15194261 (PMC10574031; doi:10.3390/nu15194261)
Supplement: Supplementary file 1 [file nutrients-15-04261-s001.zip › nutrients-2584130-supplementary.pdf]

nutrients

MDPI

Supplementary Materials

Dys-R Questionnaire: A Novel Screening Tool for Dysbiosis Linked to Impaired Gut Microbiota Richness

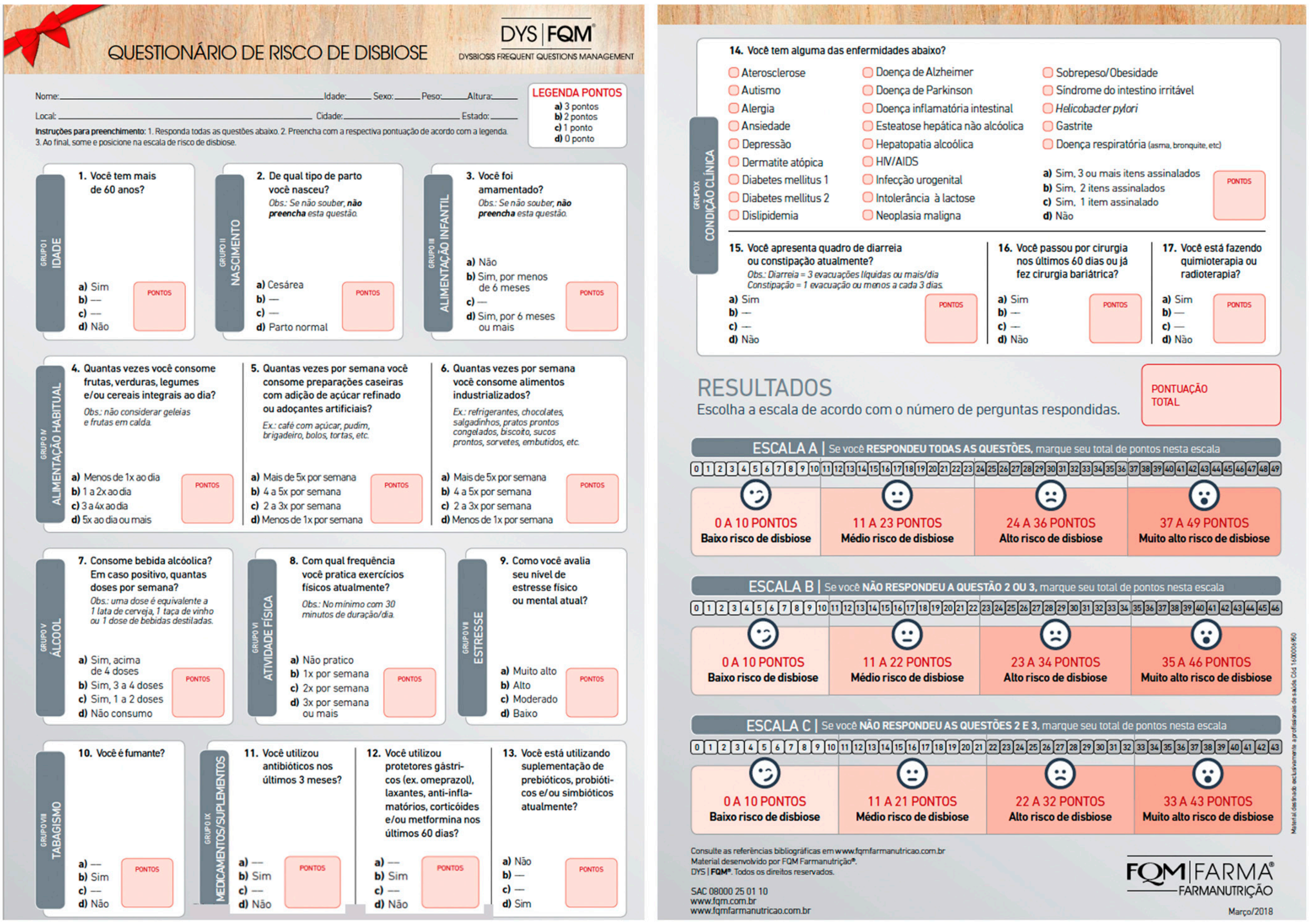

QUESTIONÁRIO DE RISCO DE DISBIOSE

DYS | FQM®
DYSBIOSIS FREQUENT QUESTIONS MANAGEMENT

Nome: ______ Idade: ____ Sexo: ____ Peso: ____ Altura: ____
Local: ______ Cidade: ______ Estado: ______

Instruções para preenchimento: 1. Responda todas as questões abaixo. 2. Preencha com a respectiva pontuação de acordo com a legenda. 3. Ao final, some e posicione na escala de risco de disbiose.

LEGENDA PONTOS
a) 3 pontos
b) 2 pontos
c) 1 ponto
d) 0 ponto

GRUPO I – IDADE
1. Você tem mais de 60 anos?
a) Sim **b)** — **c)** — **d)** Não — PONTOS

GRUPO II – NASCIMENTO
2. De qual tipo de parto você nasceu?
*Obs.: Se não souber, **não preencha** esta questão.*
a) Cesárea **b)** — **c)** — **d)** Parto normal — PONTOS

GRUPO III – ALIMENTAÇÃO INFANTIL
3. Você foi amamentado?
*Obs.: Se não souber, **não preencha** esta questão.*
a) Não **b)** Sim, por menos de 6 meses **c)** — **d)** Sim, por 6 meses ou mais — PONTOS

GRUPO IV – ALIMENTAÇÃO HABITUAL
4. Quantas vezes você consome frutas, verduras, legumes e/ou cereais integrais ao dia?
Obs.: não considerar geleias e frutas em calda.
a) Menos de 1x ao dia **b)** 1 a 2x ao dia **c)** 3 a 4x ao dia **d)** 5x ao dia ou mais — PONTOS

5. Quantas vezes por semana você consome preparações caseiras com adição de açúcar refinado ou adoçantes artificiais?
Ex.: café com açúcar, pudim, brigadeiro, bolos, tortas, etc.
a) Mais de 5x por semana **b)** 4 a 5x por semana **c)** 2 a 3x por semana **d)** Menos de 1x por semana — PONTOS

6. Quantas vezes por semana você consome alimentos industrializados?
Ex.: refrigerantes, chocolates, salgadinhos, pratos prontos congelados, biscoito, sucos prontos, sorvetes, embutidos, etc.
a) Mais de 5x por semana **b)** 4 a 5x por semana **c)** 2 a 3x por semana **d)** Menos de 1x por semana — PONTOS

GRUPO V – ÁLCOOL
7. Consome bebida alcóolica? Em caso positivo, quantas doses por semana?
Obs.: uma dose é equivalente a 1 lata de cerveja, 1 taça de vinho ou 1 dose de bebidas destiladas.
a) Sim, acima de 4 doses **b)** Sim, 3 a 4 doses **c)** Sim, 1 a 2 doses **d)** Não consumo — PONTOS

GRUPO VI – ATIVIDADE FÍSICA
8. Com qual frequência você pratica exercícios físicos atualmente?
Obs.: No mínimo com 30 minutos de duração/dia.
a) Não pratico **b)** 1x por semana **c)** 2x por semana **d)** 3x por semana ou mais — PONTOS

GRUPO VII – ESTRESSE
9. Como você avalia seu nível de estresse físico ou mental atual?
a) Muito alto **b)** Alto **c)** Moderado **d)** Baixo — PONTOS

GRUPO VIII – TABAGISMO
10. Você é fumante?
a) — **b)** Sim **c)** — **d)** Não — PONTOS

GRUPO IX – MEDICAMENTOS/SUPLEMENTOS
11. Você utilizou antibióticos nos últimos 3 meses?
a) — **b)** Sim **c)** — **d)** Não — PONTOS

12. Você utilizou protetores gástricos (ex. omeprazol), laxantes, anti-inflamatórios, corticóides e/ou metformina nos últimos 60 dias?
a) — **b)** Sim **c)** — **d)** Não — PONTOS

13. Você está utilizando suplementação de prebióticos, probióticos e/ou simbióticos atualmente?
a) Não **b)** — **c)** — **d)** Sim — PONTOS

GRUPO X – CONDIÇÃO CLÍNICA
14. Você tem alguma das enfermidades abaixo?
- Aterosclerose
- Autismo
- Alergia
- Ansiedade
- Depressão
- Dermatite atópica
- Diabetes mellitus 1
- Diabetes mellitus 2
- Dislipidemia
- Doença de Alzheimer
- Doença de Parkinson
- Doença inflamatória intestinal
- Esteatose hepática não alcóolica
- Hepatopatia alcóolica
- HIV/AIDS
- Infecção urogenital
- Intolerância à lactose
- Neoplasia maligna
- Sobrepeso/Obesidade
- Síndrome do intestino irritável
- *Helicobacter pylori*
- Gastrite
- Doença respiratória (asma, bronquite, etc)

a) Sim, 3 ou mais itens assinalados **b)** Sim, 2 itens assinalados **c)** Sim, 1 item assinalado **d)** Não — PONTOS

15. Você apresenta quadro de diarreia ou constipação atualmente?
Obs.: Diarreia = 3 evacuações líquidas ou mais/dia. Constipação = 1 evacuação ou menos a cada 3 dias.
a) Sim **b)** — **c)** — **d)** Não — PONTOS

16. Você passou por cirurgia nos últimos 60 dias ou já fez cirurgia bariátrica?
a) Sim **b)** — **c)** — **d)** Não — PONTOS

17. Você está fazendo quimioterapia ou radioterapia?
a) Sim **b)** — **c)** — **d)** Não — PONTOS

RESULTADOS
Escolha a escala de acordo com o número de perguntas respondidas.

PONTUAÇÃO TOTAL

ESCALA A | Se você **RESPONDEU TODAS AS QUESTÕES**, marque seu total de pontos nesta escala
0 1 2 3 … 49

0 A 10 PONTOS	11 A 23 PONTOS	24 A 36 PONTOS	37 A 49 PONTOS
Baixo risco de disbiose	Médio risco de disbiose	Alto risco de disbiose	Muito alto risco de disbiose

ESCALA B | Se você **NÃO RESPONDEU A QUESTÃO 2 OU 3**, marque seu total de pontos nesta escala
0 1 2 3 … 46

0 A 10 PONTOS	11 A 22 PONTOS	23 A 34 PONTOS	35 A 46 PONTOS
Baixo risco de disbiose	Médio risco de disbiose	Alto risco de disbiose	Muito alto risco de disbiose

ESCALA C | Se você **NÃO RESPONDEU AS QUESTÕES 2 E 3**, marque seu total de pontos nesta escala
0 1 2 3 … 43

0 A 10 PONTOS	11 A 21 PONTOS	22 A 32 PONTOS	33 A 43 PONTOS
Baixo risco de disbiose	Médio risco de disbiose	Alto risco de disbiose	Muito alto risco de disbiose

Consulte as referências bibliográficas em www.fqmfarmanutricao.com.br
Material desenvolvido por FQM Farmanutrição®.
DYS | FQM®. Todos os direitos reservados.
SAC 08000 25 01 10
www.fqm.com.br
www.fqmfarmanutricao.com.br

FQM | FARMA — FARMANUTRIÇÃO

Março/2018

Supplementary Figure S1. Questionnaire registered in Brazil at the National Institute of Industrial Property (INPI) by the Farmoquímica® Laboratory called Dysbiosis Frequent Questions Management (DYS/FQM)

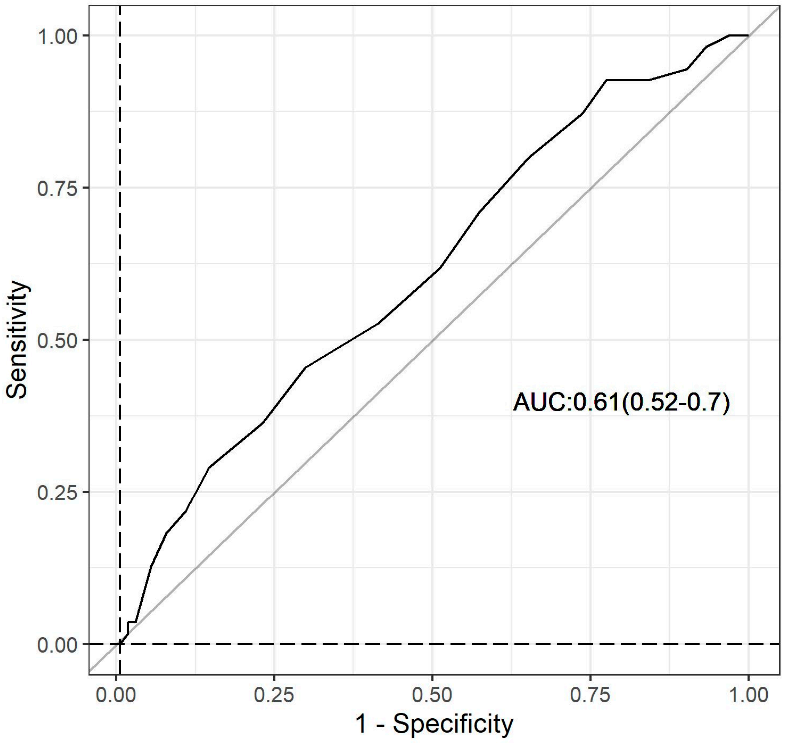

Supplementary Figure S2. Receiver Operating Characteristic (ROC) curve of the Dysbiosis Frequent Questions Management (DYS/FQM)
